# Effectiveness of Chinese pine (*Pinus tabulaeformis*) plantation at reducing runoff and erosion rates in Anjiagou Watershed in Semi-arid Region of Gansu, China

**DOI:** 10.1371/journal.pone.0271200

**Published:** 2022-07-08

**Authors:** Yanting Hu, Qing Tian, Jinxia Zhang, Glenn Benoy, Nasem Badreldin, Zisheng Xing, Zhuzhu Luo, Fu Zhang

**Affiliations:** 1 Faculty of Forestry, Gansu Agricultural University, Lanzhou, Gansu, China; 2 Gansu Provincial Key Laboratory of Arid Land Crop Science, Lanzhou, Gansu, China; 3 College of Water Conservancy and Hydropower Engineering, Gansu Agricultural University, Lanzhou, Gansu, China; 4 Faculty of Forestry and Environmental Management and the Canadian Rivers Institute, University of New Brunswick, Fredericton, New Brunswick, Canada; 5 Department of Soil Science, University of Manitoba, Winnipeg, Manitoba, Canada; 6 Faulty of Forestry and Environmental Management, University of New Brunswick, Fredericton, New Brunswick, Canada; Jinan University, CHINA

## Abstract

China’s Loess Plateau regions have experienced severe soil erosion for many decades due to fragmented landscapes, steep slopes, and concentrated rainfall storm-events. Restoring sub-optimal or marginal farming fields, mostly on steep, hilly terrain, to plantation forests has been a long-standing strategic policy in China aimed at rehabilitating degraded environments and reducing soil and water erosion. While there are numerous studies that have focused on the effects of forests at controlling soil erosion at relatively short time scales, few have addressed longer-term effects of plantation forests on reducing runoff and the mechanisms that inhibit erosion. Chinese pine (*Pinus tabulaeformis*) has been widely planted in abandoned or reclaimed lands that were formerly farmed in Northwest China; however, there is limited knowledge about the effectiveness of the tree species at reducing soil and water erosion. In this study, we examined reduction rates of runoff and erosion by Chinese pine plantation in comparison with agricultural land as a control (i.e., wheat, a dominant agricultural commodity in the region), based on long-term monitoring of modified standard erosion plots with slopes of 10°, 15°, and 20°. Results showed that as the slope of the land increased, rates of erosion increased for both plantation and agricultural land use. However, the runoff and soil erosion rates under Chinese pine plantation forest were about 11% and 60% lower, respectively, than those under agricultural land use of the same slope. Scaling with the slope, the highest reduced runoff and erosion rates by Chinese pine forest were 17% and 72%, respectively, on 20° slope. Also, it was found that runoff rates from the forested land were positively related to erosive rainfall (i.e., rainfall when runoff generated), and varied with forest canopy coverage. The rates of runoff and erosion can be well model led with multiple regression models. Taken together, this study provides insight into the importance and potential of Chinese pine plantations in the conservation of soil and water in China’s Loess Plateau.

## Introduction

Water runoff and soil erosion remain a great land management challenge due to their negative impacts such as degraded high-quality land, nutrient loss and reduced field fertility, and deterioration of aquatic ecosystems [1–4]. Substantial effort has been committed to recovering degraded land at regional and national scales, with success stories documented around the world. For example, according to National Bureau of Statistics of China, the eroded area in China has decreased from 394.9×10^4^ km^2^ in 2011 to 271.1×10^4^ km^2^ in 2019, of which 9% reduction can be attributed to remarkable afforestation efforts. The reported erosion rate of 0.9 t ha^-1^yr^-1^in 2019 in China [5] is almost two-thirds lower than the global average of soil erosion rate of 2.4 t ha^-1^ yr^-1^ [6]. However, behind these statistics, there is still an imperfect understanding of the factors that drive soil erosion processes and rates–they are at once modified by anthropogenic activities such as land management, land use change, runoff control, and by natural factors such as climate, topography, and natural vegetation. Therefore, there is still a need to assess the effectiveness of various land management practices on soil erosion and their controlling mechanisms under varying environmental conditions [4,7,8].

It has been shown that soil erosion rates under typical farming are one to two orders of magnitude higher than those observed under the original vegetation cover [9]. With increased demand for global food production, natural vegetated land continues to be exploited around the world (e.g. land conversion of steep sloping area in some areas of China). In general, this land transformation leads to reduction in soil fertility, environmental deterioration, impairment of water quality, and damage to aquatic habitats. However, improvements in the efficiency of food production technologies and its reduction on overall agricultural demand for resources, means that there are opportunities for the restoration of degraded lands to more natural environments, which has become a high-priority for many governments [4,10–12]. For example, in the Arid and Semi-arid regions of China, various large scale vegetation restoration projects have been proposed and implemented to reduce or control water runoff and soil erosion, particularly through significantly increasing vegetation cover in the lower, poorly vegetated areas (< 60%), which has been shown to potentially decrease runoff, soil loss, and flooding from high river levels and flows [13–15]. However, the effectiveness of runoff reduction brought about by vegetation restoration, such as afforestation, can be complex and vary spatially and temporarily. Therefore, a scientifically based assessment of the effects of the land restoration activities on hydrological processes and environmental conditions remains an important area of study, especially in the context of a changing climate and associated impacts on watershed hydrology and vegetation dynamics [16,17].

Soil erosion in China’s Loess Plateau has been considered a serious threat to the economic development, environmental sustainability and ecological integrity of the region for decades. In 2019, the Loess Plateau’s erosion area was 21.0×10^4^ km^2^ [18], accounting for 37% of the total land area, contributing about 90% of the Yellow River sediment [19]. Since 1950, the Loess Plateau’s climate has undergone significant changes, tending to be warmer and drier [20,21], which may have contributed to the reduction of runoff. In addition, small-scale engineering measures aiming to reduce water runoff and soil erosion may have also played a critical role in reducing soil and water erosion in the Loess Plateau by changing land use patterns [22,23]. Commencing in the early 1980s, combinations of land management practices to small watersheds, including combinations of terraces, afforestation, grass planting, nature reserves, and check dam construction, were implemented and are now the primary conservation measures across the region. For example, in 1999, a comprehensive land management project of soil and water conservation was initiated in the region [24,25]. The project is an example of the ’Grain for Green’ Program (hereafter, GGP) and was designed to return hard-to-cultivate farmland to forest and grassland for reducing soil and water erosion. Implementation of the project has dramatically altered the land use structure and vegetation cover of the Loess Plateau. Compared with a 24% natural increase in the normalized difference vegetation index (NDVI) from 1982–1999 growing season in the North, the implementation of the GGP brought about significant increases in vegetation coverage as the NDVI increased to 53% during the 2000–2013 growing season, mainly in the central portion of the region [26]. This increase of 25% in the past ten years (2000–2010) [22,27], has been mostly through afforestation, grass planting, natural reservation, and other measures [8]. As a result, the overall area of sloping farmland decreased, and forest and grassland areas increased [8,28].

Many studies have demonstrated the importance of forests at reducing soil erosion. For example, a study on five types of plantations including Chinese pine forest (*Pinus tabulaeformis*), a mixing plantation forest of Chinese pine and seabuckthorn (*Hippophae rhamnoides)*, small leaf poplar plantation (*Populus simonii*), and a mixed plantation of small leaf poplar and seabuckthorn in the Semi-arid region of Liaoning, China, showed that these forests could effectively reduce runoff and soil erosion rates and that the specific runoff and erosion reduction rates under Chinese pine were 10% and 3%, respectively, compared with bare land on an annual basis [29]. Another study implemented in China’s Loess Plateau suggested that Chinese pine plantation could reduce annual runoff and soil loss by 84% and 100%, respectively, compared with agricultural land [30]. Through monitoring of plantations in China’s loess area, Pan and Shangguan [31] found that the bare land runoff velocity and sediment concentration resulting from one rainfall event could be 23.5 times of that from the Chinese pine forest. This study also showed that Chinese pine plantations could reduce runoff and soil erosion by 88% and 99%, respectively, compared with bare land [31].

Chinese pine is a popular tree species used in many plantation forests in China. It is planted on abandoned / returned sloping-farmland for the purpose of improving the ecological environment, controlling soil erosion, and reversing land desertification from past decades [32–34]. Although there have been many studies on the species, most have focused on short-term effects on runoff and sediment loss, mostly in the rocky mountainous areas of Northern China. Due to the complexity of interactions between runoff and erosion processes, and climate change, topographic conditions, slope, aspect, soil properties, and localization of precipitation, in mountainous and complex regions like the Northern Hebei Province and Northern Shaanxi, Northern China, study findings tend to have limited transferability to other regions where the soil and topographic conditions are different, such as the Arid and Semi-arid region where the current study was conducted. Consequently, long-term reduction effects of plantations on runoff and erosion in the Semi-arid region of China remains poorly understood, which is problematic for the ecological assessment of degraded land restoration and planned afforestation under the GGP.

Long-term field monitoring methods based on standard erosion plots provide controlled and valid experimental data, which can be used to analyze runoff and erosion patterns at field scale to develop mechanistic and regression models [3]. Fitted models with data from long-term monitoring can be used as theoretical support for the development of comprehensive soil and water conservation measures and their assessment. However, long-term monitoring data is often unavailable due to the need for a long-term commitment, including sustained and often high costs, and labor demands. The Anjiagou watershed observatory station in the Loess Plateau, Dingxi, Gansu, China was established in 1953, dedicated to carry out long-term soil and water erosion studies and develop suitable land management practices for the arid and semi-arid area of this region through its unique experimental design and development of conservation practices [35,36].

The long-term monitoring data collected from this site provides an opportunity to study runoff and erosion rates under Chinese pine plantation and compare them with those under agriculture. We hypothesize that soil and water erosion may be mainly determined by a defined set of key factors (i.e., land use, slope, erosive rainfall, and forest canopy coverage) at the controlled erosion monitoring site. Therefore, the objectives of this study were to: (1) analyze the effectiveness of Chinese pine plantation at reducing rates of water runoff and soil erosions and their relationships with erosive rainfall, slope, and forest canopy development, (2) compare the runoff and erosion reduction effects of the plantation with agricultural land (i.e., wheat) under different sloping conditions, and (3) develop multivariate regression models for predicting water runoff and soil erosion rates using rainfall parameters, and canopy cover for varying sloping conditions (i.e., 10°, 15°, 20°). The results from this study will provide a scientific base and useful knowledge for the assessment of ecological restoration of degraded land in the Loess Plateau of China and other similar regions with disturbed land.

## Materials and methods

### Study area

Anjiagou watershed (104°38’13”–104°40’25”E, 35°33’02”–35°35’29”N) is located in Anding District, Dingxi City, Gansu Province, China [37]. It is a third-level branch of the Zuli River, which is a first-level tributary of the Yellow River [36], covering a drainage area of 8.5 km^2^ with an above sea level (ASL) range of 1914–2231 m. The landform belongs to the fifth sub-region of the Loess Hilly and Gully Region. The climate is classified as a mid-temperate semi-arid. The monthly mean daily temperature varies from– 6.9°C in January to 19.3°C in July with an annual average temperature of 7.2°C (1981–2010 climate normal), and an annual average precipitation of 377 mm (1986–2010 climate normal). Precipitation is unevenly distributed through the year, with 79% concentrated from the May to September period mostly as heavy rainstorms. Natural vegetation can be classified as the arid forest-steppe, and the main vegetation includes Chinese pine (*Pinus tabuliformis*), locust (*Robinia pseudoacacia*), seabuckthorn (*Hippophae rhamnoides*), pea shrub (*Caragana korshinskii*), alfalfa (*Medicago sativa*), and common sainfoin (*Onobrychis viciifolia*). Various crops growing in the region include wheat (*Triticum aestivum*), potato (*Solanum tuberosum*), and peas (*Pisum sativum*), among others.

The monitored erosion plots were located in the Anjiagou runoff test site, which has a shady slope (or north facing slope) and an altitude of 2040 – 2055m. It is a certified monitoring station by China Soil and Water Conservation Bureau, typical for soil and water conservation research. Compared to standard erosion plots, the ones at this study site were designed according to the growth characteristics of Chinese pine trees over time and wheat cropping, so that a comparison of effects of Chinese pine forest and wheat on runoff and soil erosion rates can be done in the same area on an ongoing and long-term basis.

### Erosion plot setup

**Fig 1:**
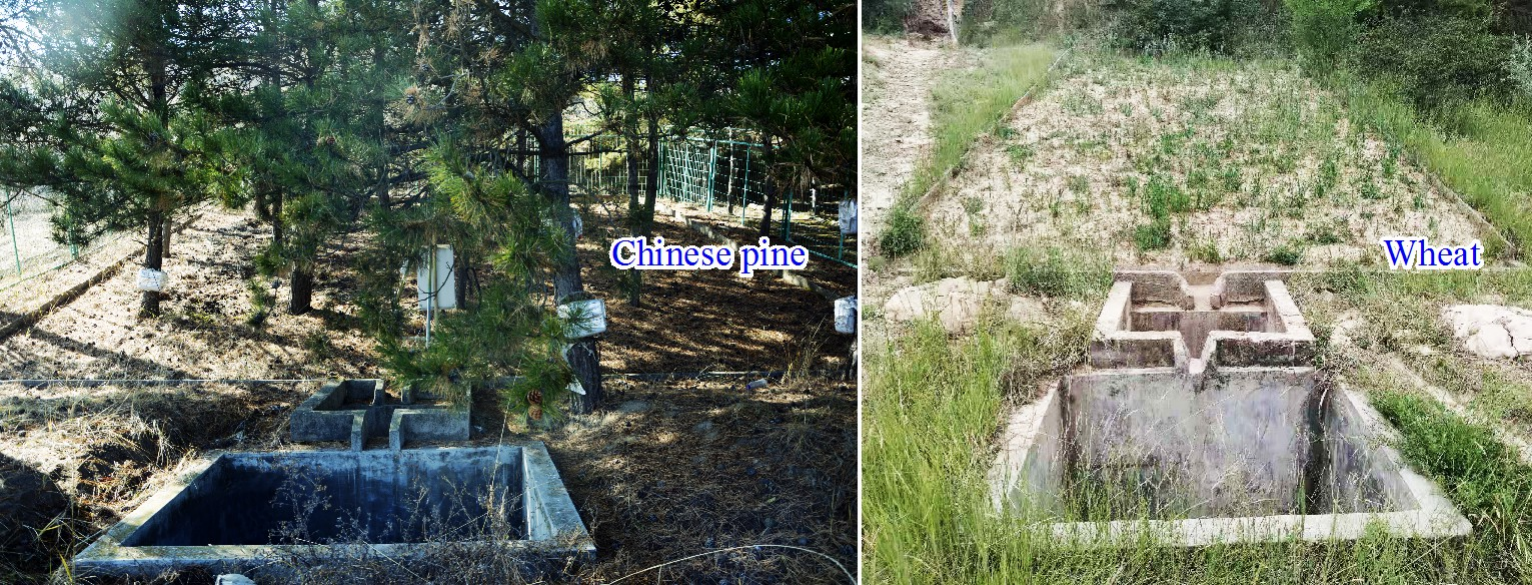
Status of the Chinese pine and wheat erosion plots in Anjiagou watershed.

The erosion plots in this study were initially established in 1986 with a plot size of 10.0 m × 10.0 m for Chinese pine plantation and 10.0 m × 5.0 m for the wheat crop (i.e. the control plots) (Fig 1), at three slopes (10°, 15°, and 20°) (Table 1). The number of wheat plots were doubled for each slope so that crop rotation could be completed on each plot, (i.e., plot 2 and 4, 1 and 5, 11 and 12 in Table 1), rotated between wheat and alfalfa (or red bean grass; *Onobrychis viciaefolia* Scop.) at least every 4 years. To increase the comparability of the research results between Chinese pine plots and crop plots, the study only counted the runoff plots with wheat years. For each plot, a concrete curb was constructed along the plot edge to enclose the plot area. There was a slot on the lower edge of each plot to lead the runoff into a 1-m^3^ erosion collection pool as shown in Fig 1.

**Table 1:** Description of erosion plots under different land uses.

Land use	Plot number	Slope (°)	Area (m^2^)	Tree age at planting (years)	Planting spacing (m×m)	Mean tree height (m)		Canopy coverage (%)	
						1986	2019	1986	2019
Chinese pine forest	8	10	100.0	8	2.0×3.0	2.4	9.2	0.4	0.9
	7	15	100.0	8	2.0×3.0	2.3	8.9	0.3	0.9
	10	20	100.0	8	2.0×3.0	2.4	9.5	0.4	0.9
Control (wheat)	2/4	10	50.0	—	—	—	—	—	—
	1/5	15	50.0	—	—	—	—	—	—
	11/12	20	50.0	—	—	—	—	—	—

Note: For control land use, two numbers in control" Plot number" indicate the rotation plot numbers between wheat and alfalfa or red bean, at least every 4 years.

### Growth of Chinese pine plantation on the modified standard erosion plots

**Fig 2:**
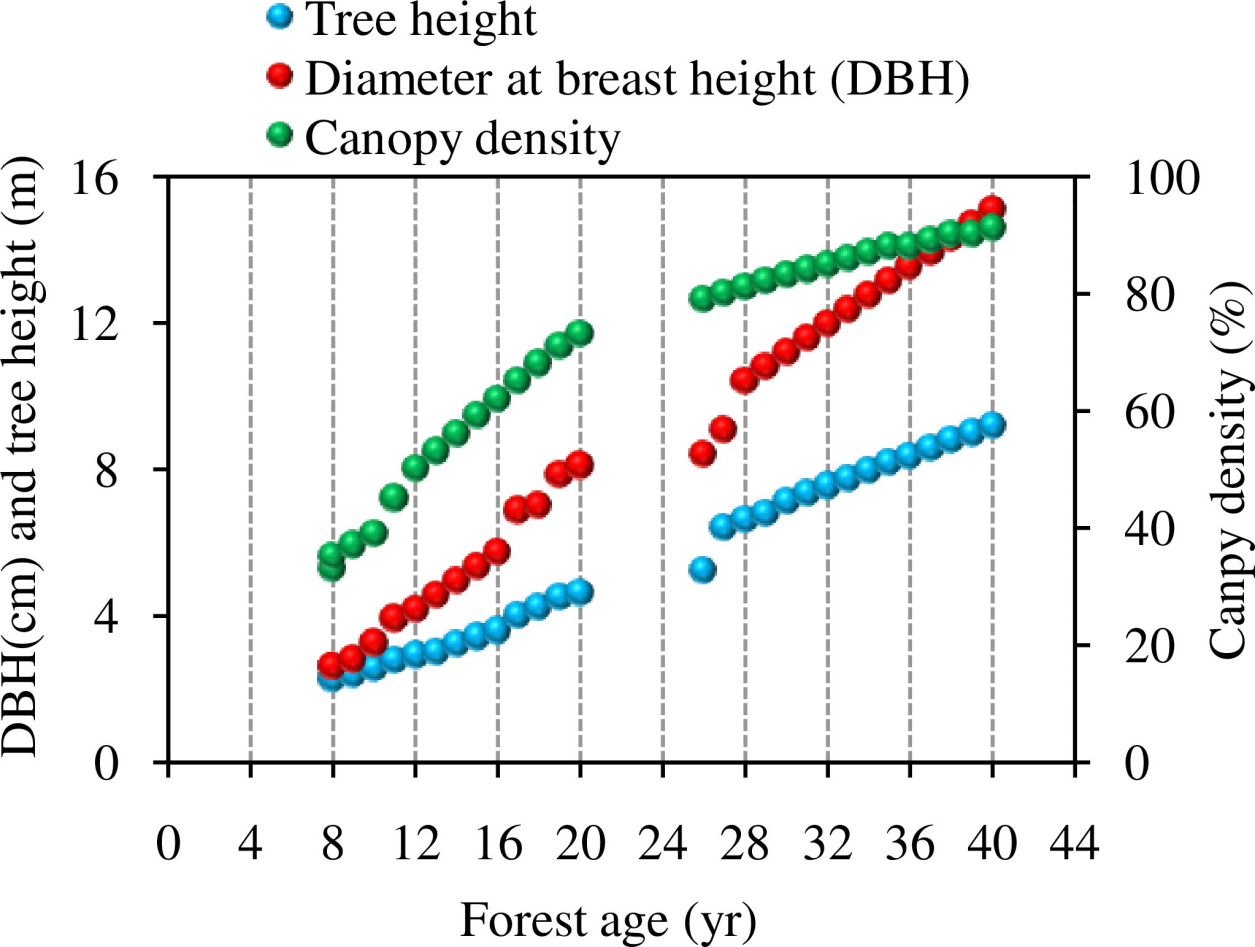
Growth of Chinese pine plantation on the study site.

In 1986 when the erosion plots were established, 8-year-old seedlings of Chinese pine were planted in erosion plots to simulate forest land use. To characterize the tree growth, tree height, diameter at breast height, and canopy coverage were measured every year from 1986 to 2019. Overall, as the forest aged, the height, diameter at breast height and canopy cover of the Chinese pine forest all showed a rapid increase from year 8 to year 26, with an increasing rate of 132% and 139% for forest height and canopy cover, respectively. However, from year 27 to 40, the tree height increase and canopy expansion rates slowed down to 44% and 14%, respectively. The average increase of the diameter at breast height before year 28 was 297% and 40% after year 28 (Fig 2). The crown increase slowed down after year 21.

### Data collection and processing

Total rainfall, erosive rainfall, average rainfall intensity, and runoff and erosion amounts were collected at the different slopes (10°, 15°, and 20°) from 1986–2019 for the Chinese pine plantation plots and from 1986–1992, 2007–2019 for the wheat (control) plots. The runoff and erosion amounts measured from each plot were standardized as runoff rate (m^3^ ha^-1^) and soil erosion rate (t ha^-1^) for the convenience of quantitative analysis and comparison between different plots [7,38].

### Statistical analysis and regression modeling

Two-factor analysis of variance (ANOVA) was used to investigate the variability of runoff and erosion rates with land use and slope. Two data processes were used to validate the comparison. First, the raw rainfall observations were binned based on rainfall amount. Erosive rainfall is defined as a rainfall (from the start to end of a rainfall) which could generate runoff. The erosive rainfall in the study area was mainly distributed in the range of moderate rain (10.0–25.0 mm) [39]. In order to study the evolution of soil erosion in this range, the erosive rainfall was subdivided according to 5.0 mm intervals: 0.0–5.0 mm, 5.0–10.0 mm, 10.0–15.0 mm, 15.0–20.0 mm, 20.0–25.0 mm, 25.0–30.0 mm, 30.0–35.0 mm, 35.0–40.0 mm, 40.0–45.0 mm, and 45.0–80.0 mm for a total of 10 bins (Table 2), and correspondingly, the observed runoff and erosion rates were summarized into a corresponding bin on a yearly basis. Second, the observations were averaged based on erosive rainfall event per year, which is called event-based analysis in this study. Each erosive rainfall event was considered as an individual data point to calculate averages of runoff rate and erosion rate on a yearly basis.

**Table 2:** Summary information for erosive rainfall observed on the erosion plots at different slope under different land use.

Land use	Slope (°)	Frequency of erosive rainfall (number of rainfall)									
		(0.0–5.0]	(5.0–10.0]	(10.0–15.0]	(15.0–20.0]	(20.0–25.0]	(25.0–30.0]	(30.0–35.0]	(35.0–40.0]	(40.0–45.0]	(45.0–80.0]
		Ⅰ	Ⅱ	Ⅲ	Ⅳ	Ⅴ	Ⅵ	Ⅶ	Ⅷ	Ⅸ	Ⅹ
Chinese pine plantation	10	5.0	29.0	50.0	45.0	39.0	22.0	16.0	8.0	10.0	5.0
	15	5.0	30.0	51.0	45.0	39.0	22.0	16.0	8.0	10.0	5.0
	20	5.0	28.0	51.0	44.0	37.0	23.0	16.0	8.0	10.0	5.0
Control (wheat)	10	5.0	17.0	32.0	29.0	27.0	16.0	10.0	5.0	5.0	5.0
	15	5.0	14.0	31.0	31.0	25.0	15.0	11.0	6.0	6.0	5.0
	20	5.0	17.0	33.0	31.0	26.0	17.0	11.0	6.0	6.0	5.0

Both approaches provided valid summaries of runoff and erosion rate. A linear or non-linear model was used to compare the effects of land use, canopy coverage, and slope condition on runoff and erosion rates.

The non-linear model (i.e., power function) was used to analyze the relationship between runoff rate, erosion rate and individual explanatory variables (Eq 1), and a multiple linear regression model was used to develop a model for predicting runoff rate and erosion rate by combining all potential explanatory variables [36,40] (Eq 2):

$$Y=aX^{b}$$ (1)

Where: *Y* is the dependent variable (i.e., runoff rate, erosion rate), *X* is the independent variable (e.g., erosive rainfall, rainfall intensity, canopy coverage), and *a* and *b* are constants.

The multiple linear regression model is described as follows:

$$\ln(Y^{'})=\ln a+b_{1}\ln(X_{1})+b_{2}\ln(X_{2})+\cdots +b_{i}\ln(X_{i})+\varepsilon$$ (2)

Where: *Y’* is the dependent variable, *X*_*i*_ is the explanatory variable, and *b*_*i*_ is the slope coefficients for each explanatory variable, and ɛ is the error term.

Multiple explanatory variable *X*_1_, *X*_2_,…, *X*_*i*_ combinations explain the variance of *Y’*, which is the proportion of the total variance, represented by *r*^*2*^ coefficient of determination [41]. Since *r*^*2*^ only considers the variance and does not account for the degree of freedom, to compare different models involving varying numbers of explanatory variables with *r*^*2*^ therefore can be problematic. To provide corrections to *r*^*2*^ based on the degree of freedom, various adjusted coefficients of determination were considered [42,43]. The Ezekiel’s adjusted coefficient of multiple determinations is used in this study, which is as follows [44]:

$$r_{a}^{2}=1-(1-r^{2})\frac{n-1}{n-k-1}$$ (3)

Where: $r_{a}^{2}$ is the adjusted coefficient of determination, *r*^2^ is the coefficient of determination, *n* is the number of sample observations, and *k* is the number of observation constraints. The percentage conversion of $r_{a}^{2}$ is normally considered the explained percentage of the total variance of dependent by the explanatory variables.

All statistical analyses were completed using SPSS20 (SPSS Ltd., USA).

## Results

### Annual rainfall and average rainfall intensity during the study period

**Fig 3:**
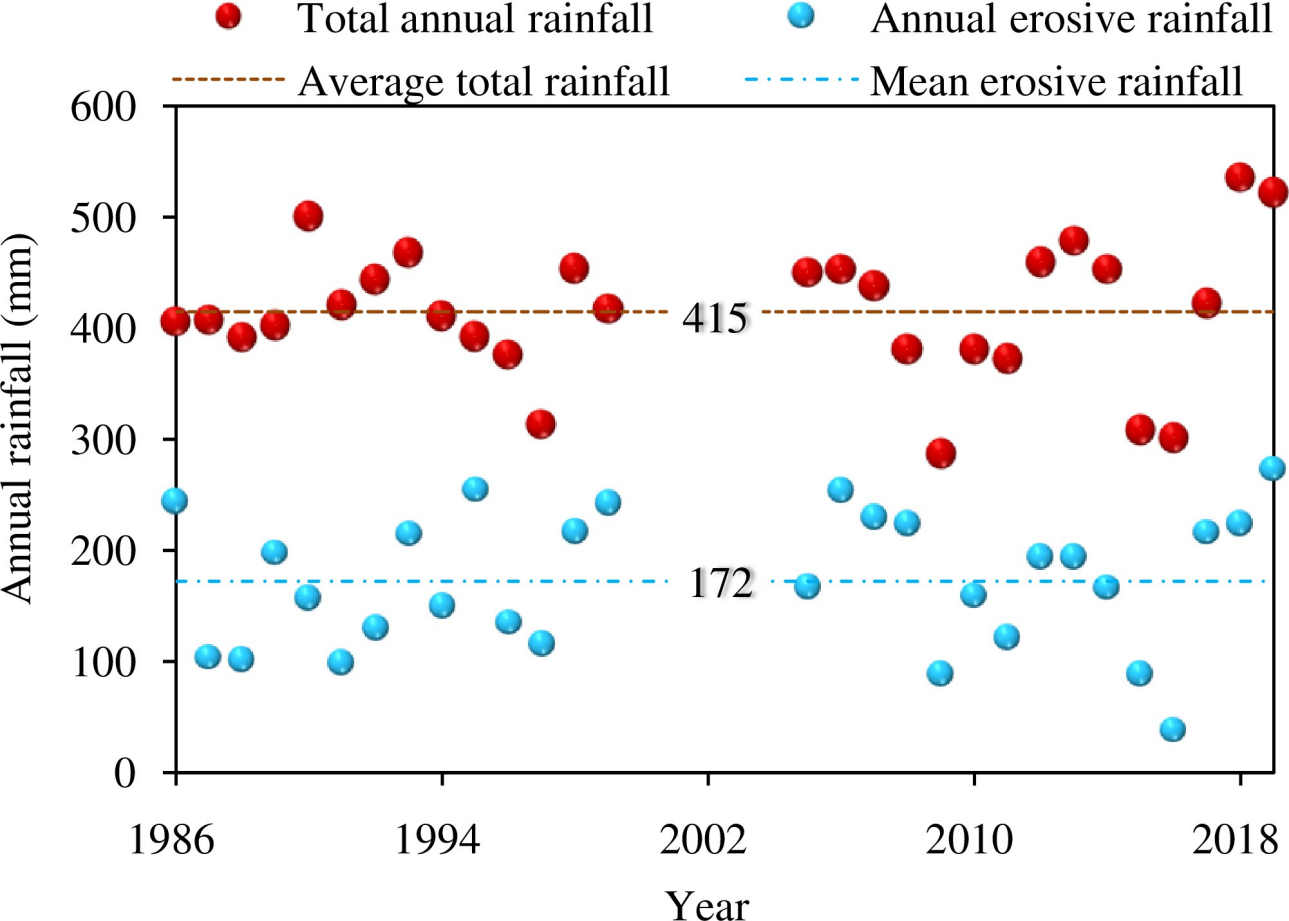
Rainfall characteristics during the study period.

In this study, the portion of annual erosive rainfall out of total rainfall ranged from 13% to 65% of total annual rainfall (Fig 3). During the observation period (1986–2018), total annual rainfall varied from a low of 286.0 mm in 2009 to a high of 535.1 mm in 2018, with an average of 414.8 mm (Fig 3). The total May-September rainfall varied from 208.0 mm in 2016 to 430.0 mm in 2018. The erosive rainfall in May-Sep varied from 38.0 mm in 2016 to 272.6 mm in 2019. The average erosive rainfall varied from 4.0 mm to 57.7 mm with an average of 19.9 mm event^-1^. While the maximum 30-min rainfall averaged 7.7 mm with a mean intensity of 7.8 mm h^-1^, the annual mean rainfall amount of 19.9 mm per event resulted in fairly high runoff and erosion due to the steep slopes and high erodibility typical of Loess Plateau that characterizes this region.

**Fig 4:**
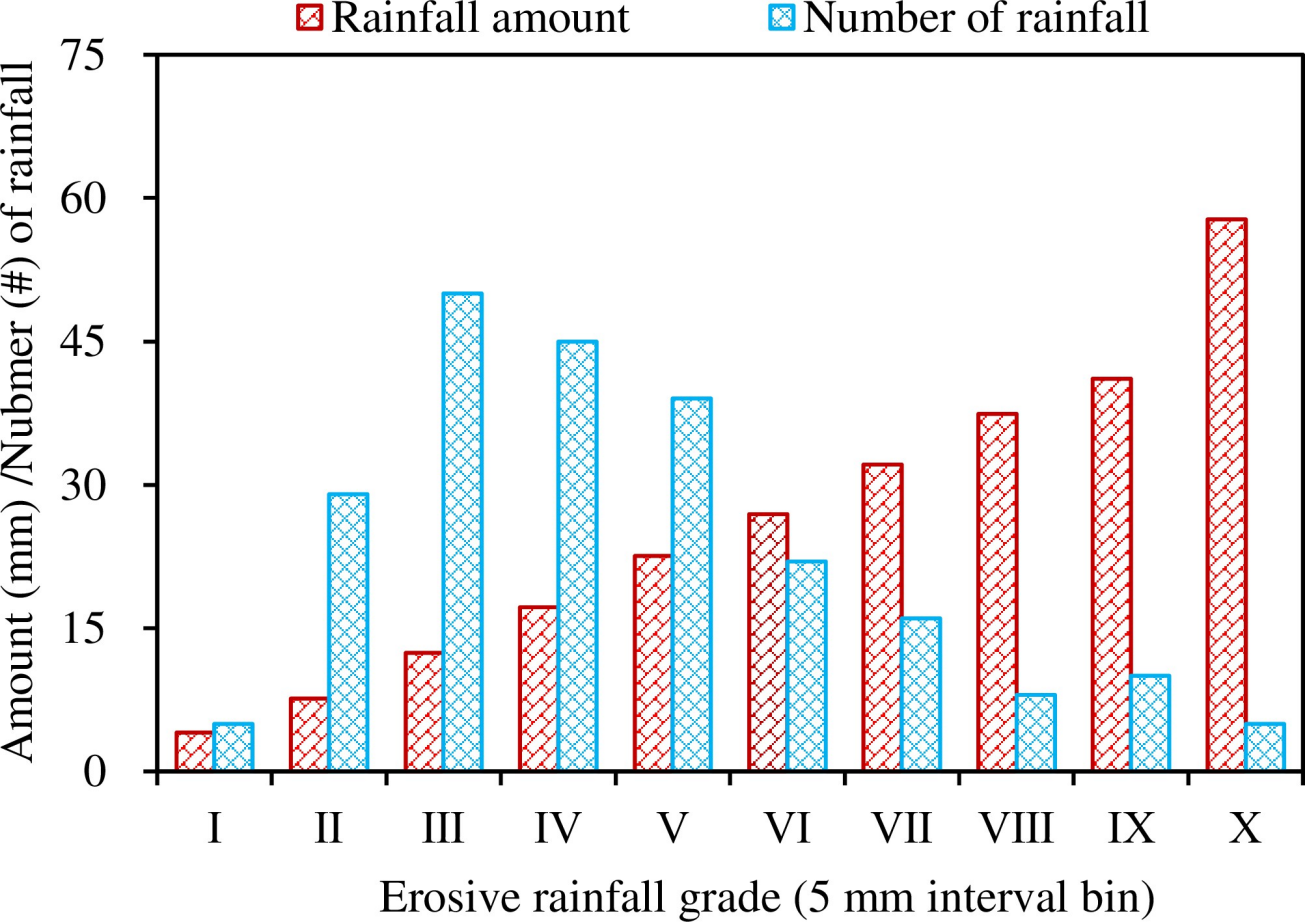
Erosive rainfall at the study site. I, II,…, X represents binned rainfall interval 0.0–5.0 mm, 5.0–10.0 mm,…, 45.0–80.0 mm, respectively.

**Fig 5:**
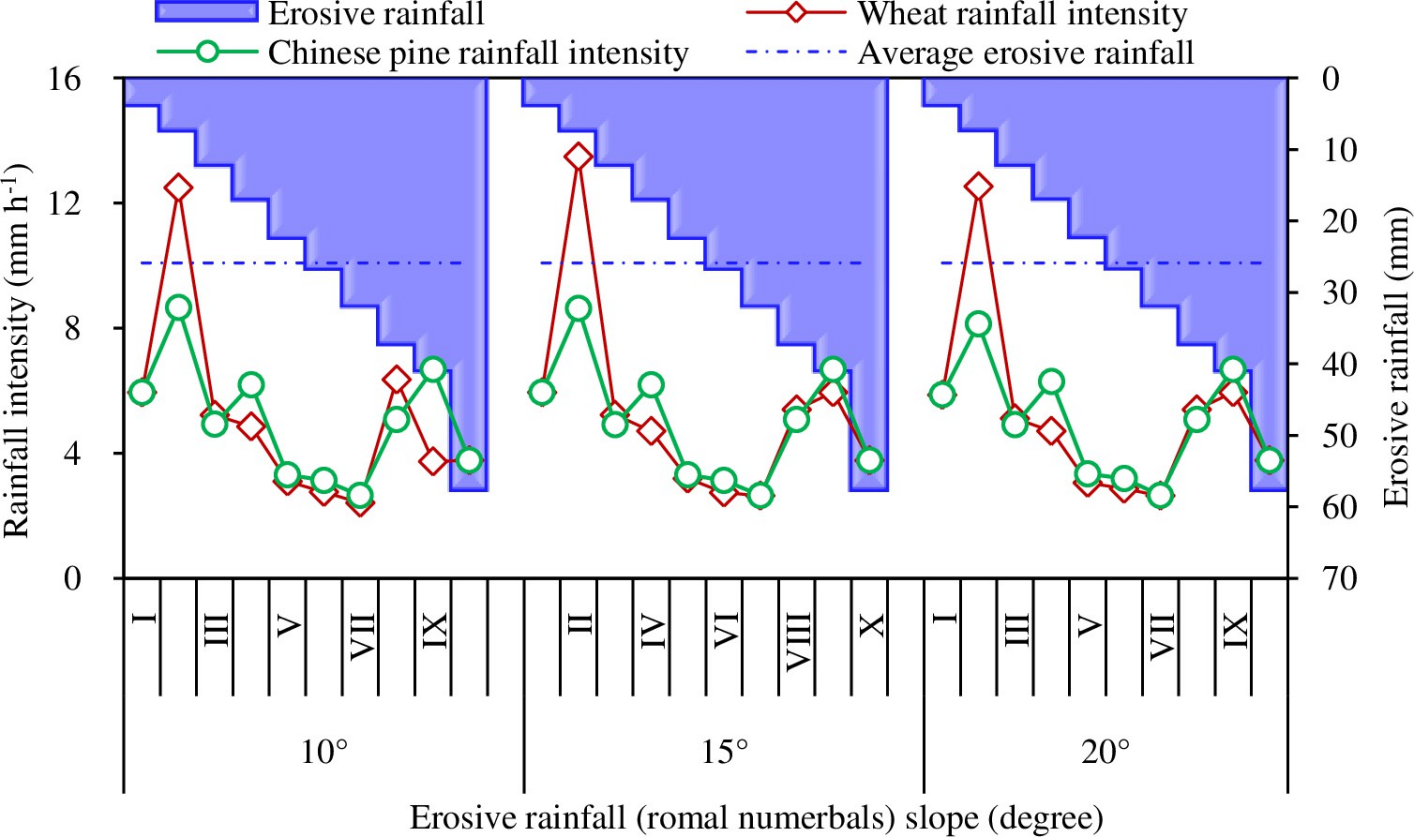
Distribution of erosive rainfall binned at 5 mm intervals. I, II,…, X represents binned rainfall interval 0.0–5.0 mm, 5.0–10.0 mm,…, 45.0–80.0 mm, respectively.

When erosive rainfall was binned at intervals of 5 mm, it was found that mean rainfall intensity varied with different erosive rainfall grades without any apparent pattern. Mean rainfall intensity was > 4.7 mm h^-1^ in grade I, II and IX, compared with other classes (< 4.7 mm h^-1^), which may mainly reflect unpredictable rainfall patterns from year to year. As shown in Fig 4, the frequently high levels of rainfall events were at erosive rainfall grade III, IV, V or rainfall amounts of 10.0–15.0 mm, 15.0–50.0 m, 20.0–25.0 mm, which corresponded to lower average rainfall intensities. In addition, erosive rainfall was identical for the two land uses, 25.9 mm for Chinese pine plots and wheat plots (Fig 5).

### Runoff and erosion rate changes

**Fig 6:**
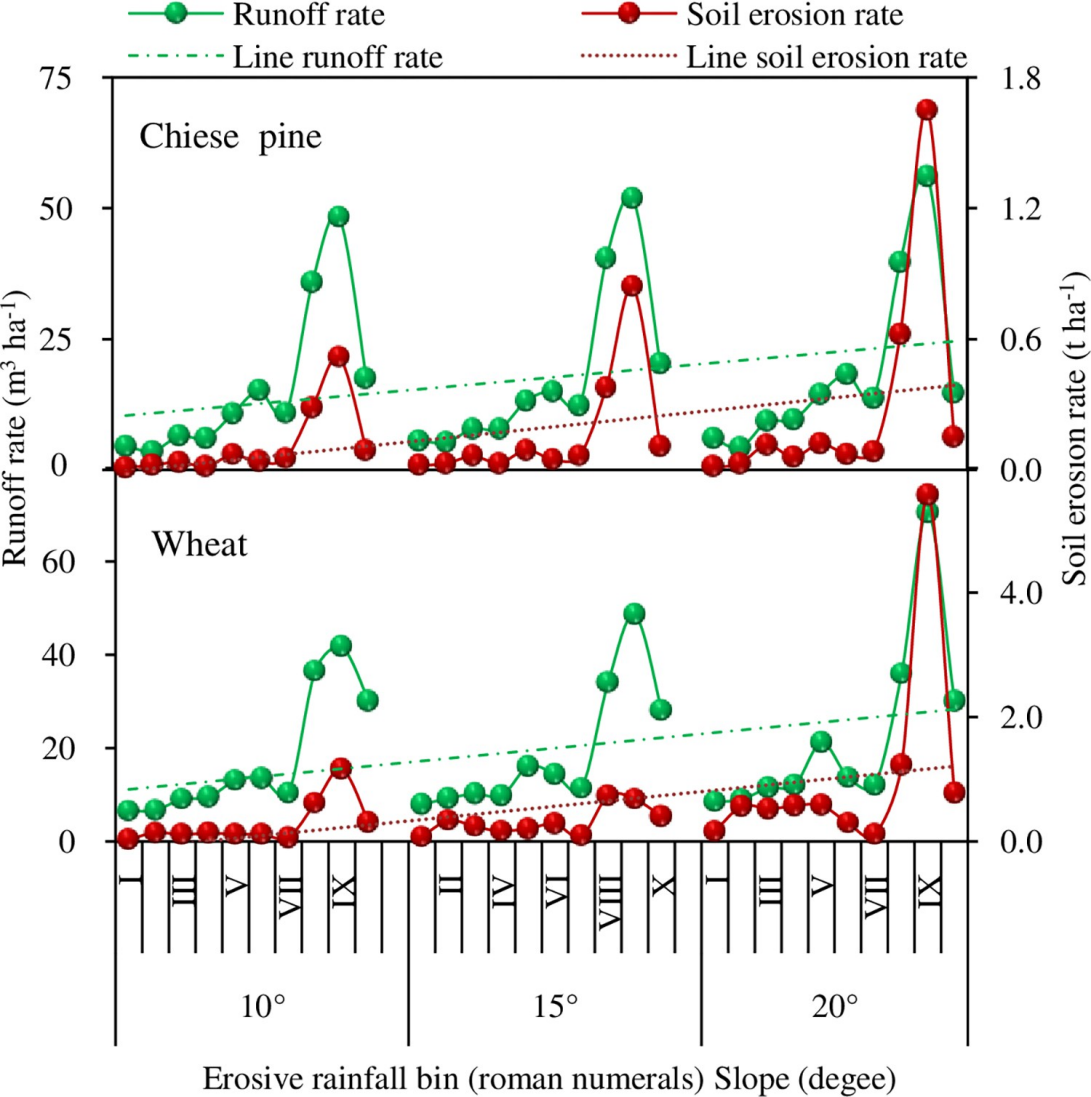
Variations of runoff and soil erosion rate at different slope. I, II,…, X represents binned rainfall interval 0.0–5.0 mm, 5.0–10.0 mm,…, 45.0–80.0 mm, respectively.

(1) Change in runoff rate. Runoff rates were found to vary with plot slope, land use, and erosive rainfall levels. For a given erosive rainfall condition, with the increase of slopes from 10° to 15° to 20°, the runoff rate increased from 15.8 m^3^ ha^-1^ to 18.6 m^3^ ha^-1^ for the Chinese pine plots and from 17.7 m^3^ ha^-1^ to 22.4 m^3^ ha^-1^ for wheat plots. The runoff rate for wheat plots was significantly higher than that from the Chinese pine plots (Table 3, *P* < 0.005, and Fig 6). The runoff rate was the greatest for the erosive rainfall grade VIII and IX, which corresponded to the larger erosive rainfall and higher mean rainfall intensity of those grades (Fig 5). This indicates that erosive rainfall, average rainfall intensity and slope, and their interaction impacted on runoff amounts.

**Table 3:** Comparison of runoff and soil erosion rate at different slope under different land use during study period (1986–2019).

Treatment	Parameter	Runoff rate (m^3^ ha^-1^)			Soil erosion rate (t ha^-1^)		
		10°	15°	20°	10°	15°	20°
Chinese pine plantation	Mean	15.8^a^	17.9^a^	18.6^a^	0.1^a^	0.2^a^	0.3^a^
	Change rate	1.0	1.1	1.2	1.0	1.5	2.6
	C.V. %	1.1	1.0	1.0	2.0	2.1	2.2
Control (wheat)	Mean	17.7^a^	19.0^a^	22.4^a^	0.3^a^	0.3^a^	1.0^b^
	Change rate	1.0	1.1	1.3	1.0	1.2	3.7
	C.V.%	1.2	1.0	1.1	1.9	1.8	2.0
Reduction rate by forest land (%)		10.5	5.7	17.2	59.6	48.1	71.9

Note: the lower case letters following a number indicate the different group compared with another slope for runoff and erosion rate separately at a *P*-level of 0.05. Summaries are based on erosive rainfall grades.

(2) Changes in soil erosion rates. Similar to the runoff variation, within the same erosive rainfall grade, the soil erosion rate increased with slope increase from 10° to 20°. However, the final increase rates varied with land use conditions (Table 3). (i) For the Chinese pine plots, the soil erosion rate increased from 0.1 t ha^-1^ to 0.3 t ha^-1^, or by 157.8%, and the soil erosion rate for the highest slope was 2.6 times that for the lowest slope. (ii) For wheat plots, the soil erosion rates were much higher from 0.3 t ha^-1^ to 1.0 t ha^-1^, an increase of 271.5% from slope 10° to 20°. The highest slope’s erosion rate was 3.7 times that of the lowest slope (Table 3). (iii) The erosion rate also varied with erosive rainfall grades with the greatest change of erosion rate with slope occurred in erosive rainfall grade VIII and IX. In other words, the changes in erosion rate corresponded to the larger classes of erosive rainfall and higher average rainfall intensity, varying with runoff rate. However, the erosion rate with smaller erosion rainfall grades (I–VII) varied less and had different changing pattern from the larger grades, varying little with runoff rate variation (Fig 6). This may indicate that the erosion rate changes were non-linearly related to slope, erosive rainfall, average rainfall intensity, and runoff rate.

(3) Interactive change of runoff and erosion with land use and slope. Based on binned erosive rainfall classes, both runoff and erosion rates appeared to show an increasing trend with slope regardless of land use, although the differences between slopes was less pronounced except for the erosion rate of the wheat plot at 20° slope. For a given slope, runoff and erosion rates of wheat plots were consistently higher than those of the Chinese pine plots. From 10° to 15° to 20°, the runoff rates of Chinese pine plots were lower than those of wheat plots by 1.9 m^3^ ha^-1^, 1.1 m^3^ ha^-1^, 3.9 m^3^ ha^-1^, respectively, while the erosion rates were lower by 0.2 t ha^-1^, 0.2 t ha^-1^ and 0.7 t ha^-1^, respectively (Table 3).

Overall, it was found that (i) for slope changes of 10° to 15° in Chinese pine plots, the runoff rate increased by 13% and erosion rate increased by 47%, and for slope changes of 15° to 20°, the rates increased by 3.8% and 75%, respectively; (ii) For the wheat plots, increasing slopes from 10° to 15°, the runoff and erosion rates increased by 7% and 15%, respectively, and from 15° to 20° they increased by 18% and 223%, respectively; and the increase in runoff and erosion rates of the wheat plots being much greater than those of the Chinese pine plots; (iii) The differences in erosion rate increase between the two land uses were much larger than that in runoff rate for a given slope; and (iv) The slope increase had a greater impact on the erosion rates in the wheat plots than in the Chinese pine plots (Fig 6).

With rainfall-event-based analysis, it was found that (i) the mean runoff rate of the wheat plots increased with increasing slope (Table 4). The runoff rate at slope 20° was significantly higher by 14–36% than those for the other slopes of the wheat plots and all Chinese pine plots. The runoff rate of the wheat plots appeared to be lower by 13% for the 10° slope than for the 15° slope, although their difference was not statistically significant. Similarly, the runoff rate from Chinese pine plots showed a decreasing trend as slope decreased. This suggests that the slope was a determining factor of runoff rate for wheat and Chinese pine plots; (ii) The mean erosion rate showed a systematic decreasing trend from high slope to low slope for each land use while the mean erosion rate of the Chinese pine plots at the highest slope was lower than that of the wheat plots at the lowest slope. The mean erosion rate for the 20° slope at the wheat plot was higher than any other plots by 61 to 91%. Similar to runoff rate, slope had a greater impact on the erosion rates of the wheat plots than on Chinese pine plots (Table 4).

**Table 4:** Runoff and erosion rate variation with land use and slope during the study period (1986–2018) based on an annual average of rainfall events, compared at *P* = 0.10 level.

Land use	Slope (°)	Mean runoff (m^3^ ha^-1^)	LSD	Reduction (%)	Mean erosion rate (t ha^-1^)	LSD	Reduction (%)
Wheat	20	16.9	A	0.0	0.7	a	0.0
Wheat	15	14.6	Ab	13.5	0.3	b	60.8
Wheat	10	13.2	Ab	21.9	0.2	b	74.3
Chinese pine	20	13.3	Ab	21.2	0.2	bc	73.0
Chinese pine	15	12.6	Ab	25.4	0.1	bc	86.5
Chinese pine	10	10.9	B	35.6	0.1	c	90.5

Note: The missing years due to other crop growth during the study period were excluded from this analysis for comparability between Chinese pine and wheat plots. Different letters in the LSD column indicate the significant statistical homogeneous group based on Least Square Difference (LSD) analysis.

Both the binned and even-based analyses obtained similar results.

### Variation in runoff and erosion rate with land use, rainfall intensity and their interactions

This study found that runoff and erosion rates varied greatly with different rainfall grades, with complex interactions between land use and rainfall intensity (Fig 6). The average intensity of the rainfall grade II was the highest (Chinese pine 8.6 mm h^-1^, wheat 12.5 mm h^-1^) regardless of land use and slope conditions (Fig 5). However, as shown in Fig 6, the corresponding runoff rate, and erosion rate were all small, mainly due to the low total erosive rainfall amount (8 mm). With an increase in the rainfall grade, the runoff rate and erosion rate showed increasing trends (Fig 6), varying significantly with land use, but only slightly with different slopes. Compared with other grades, grade VII had the smallest average rainfall intensity (2.6 mm h^-1^) regardless of slope (Fig 5), and reduced runoff rates, and erosion rates (Fig 6), likely because of the low antecedent soil moisture (about 10%) recorded in this erosive rainfall grade.

The observed variability of runoff and erosion rates with slope and rainfall grades may have been attributed to the number of rainfall events. As expected, the middle grades of rainfall range occurred more frequently than very heavy rainfall grades. Mean rainfall may not adequately represent the distribution of rainfall patterns, and skew results. As shown in Fig 4, for example, the erosive rainfall grades II, III, IV, and V were the most frequently observed in this region and may be critical when accounting for the variations in the runoff and erosion. Given the fact that the highest rainfall intensities were observed in the erosive grades II, III, IV, and IX, unexpectedly, these grades with higher rainfall intensities were not closely aligned with the trends of heavy runoff and high erosion rate. This suggests that the impacts of the other factors (e.g. rainfall duration, antecedent soil moisture, canopy condition when raining) may also affect the runoff and erosion rates. These interacting factors led to some non-linearity between the rates of runoff and erosion with erosive rainfall, particularly for wheat plots.

### Effectiveness of Chinese pine plantation on soil and water conservation

Chinese pine plots showed a systematic reduction in runoff and erosion rates compared with the wheat plots for each slope condition (Table 3). Statistically, the erosion rates of Chinese pine plots for the plot slopes 10°, 15°, and 20° were lower by 60%, 48%, and 72% than those of the wheat plots (Table 3). Similar patterns of runoff rates between Chinese pine plots and wheat plots were observed. Runoff rates of forest plots were 10%, 6%, and 17% lower than those of wheat plots for slope 10°, 15°, and 20°, respectively. The differences in the runoff and erosion rates between Chinese pine and wheat plots may mainly be attributed to the differences in plant canopy development characteristics over time. In the study area, the wheat plots were seeded in late February to early March and harvested in mid-to-late August. Therefore, a rainfall with the same amount of erosive rainfall could lead to higher runoff and erosion rate at the early stage of the wheat crop than middle and late-stage or after harvest, which resulted in more considerable non-linear variation for wheat plots within a growing season. In contrast, for Chinese pine plots, the canopy cover grew every year and leaf renewal was not noticeable normally, and interception to raindrops increased year by year, which could substantially reduce rainfall impacts on soil. Simultaneously, there was no soil cultivation under the forest canopy, and less disturbance to soil could maintain the relationship between canopy coverage and soil and water erosion effects.

### Regression analysis

Pearson correlation analyses showed that runoff and erosion rates were correlated with several factors (erosive rainfall, rainfall intensity, canopy coverage, land use, slope (Table 5). Of the factors, erosive rainfall was positively correlated (*P* < 0.05) more with runoff rate than with erosion rate, varying with land use and slopes. For Chinese pine plots, the runoff rate and erosion rate were not significantly correlated to slope and canopy cover (*P* > 0.05), but more or less explained by them with higher coefficients for erosion rate at the 20° plots than those at the 10° and 15°; A close-up study revealed that under high coverage (> 64%), heavy and long-duration (> 10 hrs) rain conditions (VI > 25 mm erosive rainfall) (Fig 5), the rain drop interception capability of the tree canopy is higher at the initial stage of the rainfall event. However, once the maximum holding capacity of the canopy was reached, the concentrated rain may down-pour to the soil surface which has already been gradually wet by rain drops through the canopy, which might show as an increased rainfall amount or intensity increase. When the soil was completely wet and fully saturated, the sustained rain could result in greater runoff and erosion rates (Fig 6) which may lead to a false positive relationship between runoff, erosion and erosive canopy coverage. However, when the canopy coverage was low, the accumulative effect of rainfall by canopy was minor and a weak negative correlation between rainfall and erosion or runoff rate should be expected due to the canopy interception. For wheat plots with different slopes, both the rates of runoff and erosion were significantly positively correlated with erosive rainfall.

**Table 5:** Pearson correlation among different parameters describing soil and water erosion from Chinese pine and wheat plots.

Land use	Slope												
	10°					15°				20°			
Chinese pine		*R*_*f*_	*C*_*d*_	*R*_*i*_	*R*_*r*_	*R*_*f*_	*C*_*d*_	*R*_*i*_	*R*_*r*_	*R*_*f*_	*C*_*d*_	*R*_*i*_	*R*_*r*_
	*C*_*d*_	0.37	1.00			0.38	1.00			0.40	1.00		
	*R*_*i*_	-0.43	-0.14	1.00		-0.43	-0.15	1.00		-0.42	-0.16	1.00	
	*R*_*r*_	0.65*	0.44	0.04	1.00	0.66*	0.46	0.05	1.00	0.57*	0.50	0.08	1.00
	*S*_*er*_	0.53	0.43	0.19	0.97**	0.48	0.42	0.23	0.95**	0.45	0.43	0.28	0.95**
Wheat (Control)	*R*_*i*_	-0.49	—	1.00		-0.44	—	1.00		-0.44	—	1.00	
	*R*_*r*_	0.80*	—	-0.22	1.00	0.74*	—	-0.09	1.00	0.64*	—	-0.05	1.00
	*S*_*er*_	0.55	—	-0.08	0.90*	0.56*	—	0.18	0.89*	0.40	—	0.12	0.94**

Note

*, ** indicate significant at *P* < 0.05 and *P* < 0.01 levels, respectively. *R*_*r*_ = runoff rate (m^3^ ha^-1^), *S*_*er*_ = soil erosion rate (t ha^-1^), *R*_*f*_ = erosive rainfall (mm), *R*_*i*_ = rainfall intensity (mm h^-1^), *C*_*d*_ = the canopy coverage (%).

(1) Simplified regression models. To further explore the impacts of various factors on runoff and erosion rates, a simplified regression was fitted to the data for Chinese pine plots and wheat plot to examine the individual relation of the factors with runoff and erosion rates (Table 6). The results showed that the erosive rainfall explained most of the total variance in runoff rate. With the increase of slopes from 10°, 15°, to 20°, the degree of explanation decreased by 64%, 63%, and 56%, respectively; in contrast, erosive rainfall explained a smaller part of the total variance in erosion rate, varying with slope as well. When the slope was 15°, only 46% of the total variance in erosion rate was explained by erosive rainfall compared with > 50% in other slope conditions (Table 6). However, the runoff rate explains most (< 88%) of the total variance in the erosion rate as expected. The impacts of canopy covers of forest plots can only be meaningful for the soil erosion rates of plots at the slope of 20°.

**Table 6:** Models for runoff rate at different slope under Chinese pine plots.

Land use	Slope	Model	Degree of freedom	r	r_*a*_^2^	F	Sig.
Chinese pine plantation	10°	$R_{r}=0.84R_{f}{}^{0.86}$	10	0.82	0.64	16.69	0.004
		$S_{er}=0.002R_{f}{}^{1.18}$	10	0.78	0.56	12.28	0.008
		$S_{er}=3.81\times 10^{-4}R_{r}{}^{1.36}$	10	0.94	0.86	59.54	0.000
	15°	$R_{r}=1.28R_{f}{}^{0.78}$	10	0.82	0.63	16.48	0.004
		$S_{er}=0.003R_{f}{}^{1.04}$	10	0.72	0.46	8.64	0.019
		$S_{er}=2.38\times 10^{-4}R_{r}{}^{1.45}$	10	0.95	0.88	77.20	0.000
	20°	$R_{r}=1.49R_{f}{}^{0.74}$	10	0.78	0.56	12.33	0.008
		$S_{er}=0.002R_{f}{}^{1.31}$	10	0.77	0.54	11.51	0.009
		$S_{er}=7.39\times 10^{-5}R_{r}{}^{1.65}$	10	0.92	0.84	46.78	0.000
Wheat (control)	10°	$R_{r}=1.79R_{f}{}^{0.69}$	10	0.83	0.65	17.87	0.003
		$S_{er}=0.01R_{f}{}^{0.91}$	10	0.69	0.41	7.17	0.028
		$S_{er}=0.001R_{r}{}^{1.40}$	10	0.88	0.75	28.36	0.001
	15°	$R_{r}=2.65R_{f}{}^{0.59}$	10	0.79	0.57	13.10	0.007
		$S_{er}=0.02R_{r}{}^{1.00}$	10	0.81	0.60	14.69	0.005
	20°	$R_{r}=2.66R_{f}{}^{0.63}$	10	0.76	0.52	10.91	0.011
		$S_{er}=0.003R_{r}{}^{1.33}$	10	0.84	0.66	18.51	0.003

Note: Only factors which have significant relation with runoff and erosion rate are listed. *R*_*r*_ = runoff rate (m^3^ ha^-1^), *S*_*er*_ = soil erosion rate (t ha^-1^), *R*_*f*_ = erosive rainfall (mm); r = correlation coefficient, F = F-test value, Sig. = significance.

For the wheat plots, erosive rainfall explained a less portion of the total variance of runoff rate (< 65%), varying with plot slope. The total variance of erosion rates was even less explained by erosive rainfall (maximum 41%, for the slope 10°), while runoff rates explained a large part of the total variance of erosion rates (< 75%) (Table 6). The total variance of the runoff rate at 20° was the least explained by erosive rainfall (52%) compared with 57% and 65% for slope 10°, 15° slope. On the other hand, no model can be fitted for erosion rate at the 15° and 20° slopes because the fitting results were not significant.

This study confirms that runoff and erosion rates are affected by many factors including some studied in this research. It is possible that additional factors may also have an effect that were not included in this study. In general, the greater the number of factors studied, the better the understanding of the mechanisms that control water runoff and soil erosion, which improves the likelihood that soil conservation problems can be solved. It is apparent that one factor could only explain relatively less of the total variances of runoff and erosion rates regardless land use and slope conditions.

(2) Multi-variat regression models. Compared with single factor regression, the multi-variate regression models provided better predictions of runoff and erosion rates. The fitting effects of erosion rate were better than runoff rate regardless of land use or slope conditions. (i) For Chinese pine plots, with the increase of slope (i.e., 10°, 15°), a combination of erosive rainfall, average rainfall intensity, and canopy cover could explain the total runoff variance by 61%, and 64%, respectively (Table 7), and there was no model fitted for the 20° slope. In contrast, the combination of erosive rainfall, rain intensity, canopy closure, and runoff rate had a higher explanation of the soil erosion rate, by 86%, 91%, and 90% for different slope (i.e., 10°, 15°, 20°), respectively, based on binned erosive rainfall (Table 7). (ii) For wheat plot, with the increase of slope (i.e., 10°, 15°, and 20°), a combination of erosive rainfall, and average rainfall intensity could explain the total runoff variance by 70%, 69%, and 62%, respectively. There is no valid model fitted for the erosion rate of wheat plots with 74–83% of variance explained by erosive rainfall, rainfall intensity, and runoff rate.

**Table 7:** Models for predicting runoff and erosion rates with different variables.

Erosive rainfall	Land use	Slope	Model	Degree of freedom	r	$r_{a}^{2}$	F
5mm binned	Chinese pine plots	10°	$\ln R_{r}=5.72+1.12\ln R_{f}+0.60\ln R_{i}-1.22\ln C_{d}$	10	0.86	0.61	5.52
			$\ln S_{er}=-12.77-0.04\ln R_{f}+0.41\ln R_{i}+1.86\ln C_{d}+1.30\ln R_{r}$	10	0.96	0.86	15.11
		15°	$\ln R_{r}=5.19+1.04\ln R_{f}+0.69\ln R_{i}-0.94\ln C_{d}$	10	0.87	0.64	6.21
			$\ln S_{er}=-13.34-0.40\ln R_{f}+0.21\ln R_{i}+1.96\ln C_{d}+1.64\ln R_{r}$	10	0.98	0.91	24.56
		20°	$\ln S_{er}=-14.19+0.32\ln R_{f}+0.96\ln R_{i}+2.41\ln C_{d}+1.25\ln R_{r}$	10	0.97	0.90	21.93
	Wheat plots	10°	$\ln R_{r}=1.33+0.86\ln R_{f}+0.49\ln R_{i}$	10	0.88	0.70	11.44
			$\ln S_{er}=-3.59+0.52\ln R_{f}+1.04\ln R_{i}+1.09\ln R_{r}$	10	0.94	0.83	16.02
		15°	$\ln R_{r}=1.90+0.77\ln R_{f}+0.58\ln R_{i}$	10	0.87	0.69	11.13
			$\ln S_{er}=-1.97+0.47\ln R_{f}+0.97\ln R_{i}+0.59\ln R_{r}$	10	0.91	0.74	9.46
		20°	$\ln R_{r}=2.04+0.82\ln R_{f}+0.64\ln R_{i}$	10	0.84	0.62	8.41
			$\ln S_{er}=-1.92+0.05\ln R_{f}+1.06\ln R_{i}+1.35\ln R_{r}$	10	0.94	0.82	14.71
Event based	Chinese pine plots	10°	$\ln R_{r}=-1.07+2.53\ln R_{f}+0.81\ln R_{i}-0.56\ln C_{d}$	19	0.66	0.32	3.84
			$\ln S_{er}=-8.90+3.15\ln R_{f}+1.20\ln R_{i}-0.67\ln C_{d}+0.79\ln R_{r}$	19	0.72	0.38	3.74
	Wheat plots	10°	$\ln R_{r}=-1.23+1.74\ln R_{f}+0.62\ln R_{i}$	19	0.57	0.23	3.76
			$\ln S_{er}=-5.07+0.82\ln R_{f}+0.82\ln R_{i}+1.04{\mathrm{lnR}}_{r}$	19	0.81	0.59	9.75
		15°	$\ln S_{er}=-0.60-0.09\ln R_{f}+1.05\ln R_{i}+0.75\ln R_{r}$	19	0.84	0.64	11.02
		20°	$\ln S_{er}=-1.06-0.68\ln R_{f}+0.68\ln R_{i}+1.47{\mathrm{lnR}}_{R}$	19	0.79	0.55	8.36

Note: Only factors which have significant relationships with runoff and erosion rate are listed at *P* < 0.05. *R*_*r*_ = runoff rate (m^3^ ha^-1^), *S*_*er*_ = soil erosion rate (t ha^-1^), *R*_*f*_ = erosive rainfall (mm), *R*_*i*_ = rainfall intensity (mm h^-1^), *C*_*d*_ = the canopy coverage (%).

In contrast, when a rainfall-event based statistical approach is used, the combination of erosive rainfall and rainfall intensity resulted indifferent degrees of predictions of runoff and erosion rates compared with the binned erosive rainfall approach (Table 7). Runoff rate models can be fitted only for the slope 10° regardless of land use, explaining less variance than the binned erosive rainfall approach. However, it can generate prediction models of erosion rates at 10° for both land uses and all slope conditions of wheat plots, explaining less than 59% of the variance in erosion rates. The performance of the event-based approach is opposite of the binned approach, each shows advantages and disadvantages. This implies that a combination of modeling of event-based and binned erosive rainfall approaches can be used for improving model predictions of runoff and erosion rates.

When introducing plot slope as a factor in the multi-variate regression, the obtained prediction models have higher adjusted determination coefficients than without slope, better model performance for erosion rate and runoff rate of Chinese pine plots than for those of the wheat plots (*P* < 0.01; Table 8). This is reasonable because as described previously, the large variation and interactions of wheat canopy cover over season with raining characteristics could best determine the impacts of slope. The combination of slope, erosive rainfall, average rain intensity, and canopy cover percentage could explain 66% of the total variance of runoff rate, and the combination of slope, erosion rainfall, average rain intensity, canopy cover percentage, and runoff rate could explain 93% of the total variance of erosion rate. The models for wheat plots performed slightly better with 71% of the total variance of runoff rate being explained by the combination of slope, erosive rainfall, and average rain intensity explained. Only 84% of the total variance of erosion rate could be explained by the combination of slope, erosive rainfall, average rain intensity, and runoff rate for the wheat plots.

**Table 8:** Predicting models of runoff and erosion rate with slope as a predict variable.

Land use	Models	Degree of freedom	r	$r_{a}^{2}$	F
Chinese pine	$\ln R_{r}=3.29+0.31\ln S+1.00\ln R_{f}+0.59\ln R_{i}-0.73\ln C_{d}$	30	0.84	0.66	15.24
	$\ln S_{er}=-14.75+0.63\ln S+0.02\ln R_{f}+0.57\ln R_{i}+2.00\ln C_{d}+1.35\ln R_{r}$	30	0.96	0.93	61.45
Wheat field	$\ln R_{r}=0.99+0.28\ln S+0.82\ln R_{f}+0.56\ln R_{i}$	30	0.86	0.71	24.96
	$\ln S_{er}=-6.35+1.35\ln S+0.25\ln R_{f}+0.93\ln R_{i}+1.13\ln R_{r}$	30	0.93	0.86	38.97

Note: Only factors which have significant relation with runoff and erosion rates are listed at *P* < 0.05. *R*_*r*_ = runoff rate (m^3^ ha^-1^), *S*_*er*_ = soil erosion rate (t ha^-1^), *S* = slope (°), *R*_*f*_ = erosive rainfall (mm), *R*_*i*_ = rainfall intensity (mm h^-1^), *C*_*d*_ = the canopy coverage (%).

## Discussion

### Runoff and erosion rates across land uses

The differences of runoff and erosion rates between different land uses may be mainly attributed to the canopy cover differences between tree plots and wheat plots. As a yearly crop, wheat canopy development occurs within a single year and experiences three major stages: tilled bare soil or before canopy forms→canopy cover→bare soil or tilled after harvest. Therefore, the runoff and erosion rates of wheat plots showed seasonal variations, in particular, as a result of rainfall events and crop canopy development. On the other hand, for Chinese pine plots, when plots were established in 1986 by planting 8-year-old seedlings, the initial coverage was about 33%, then the canopy cover gradually increased to 91% in 2019. This increase in the canopy cover of pine trees provided greater protection of soils regardless of slope, which led to average reduction 6–17% of runoff rate and 48–72% of erosion rate than wheat plots. Similar results have been reported in previous studies [45,46]; however, it is worth noting that soil texture, topographic condition, and rainfall characteristics may cause the numeric differences in runoff and soil erosion rates both spatially and temporally.

Another finding from this study was that the runoff and erosion rates from the Chinese pine plots had similar patterns to those from the wheat plots for most rainfall grades, but they were generally lower for the Chinese pine plots than for the wheat plots (Fig 6). These findings are consistent with some previous studies in the region [47,48]. This reflects the direct impacts of plant canopy coverage and rainfall patterns and their interaction on soil characteristic. This is an important finding because it suggests that forests are superior at reducing water runoff and soil erosion compared with wheat regardless of rainfall quantity, rainfall intensity and duration. Compared with the relatively less compacted, sloping farming soil layer of wheat plots, the forest floor surface of the aging Chinese pine forest, remained un-disturbed for a much longer time. Topsoil compaction was enhanced, which may produce dry soil crusts there by reducing the water infiltration capacity of soil and causing an increase in runoff for some heavy rainfall as reported in previous studies [38,49]. Meanwhile, the forest canopy had a strong inception of rain drops, which potentially reduced the detaching force of rain drips, leading to reduced runoff and erosion rate [47]. In contrast, wheat plots were cultivated or tilled every year and frequently impacted by the seasonality of crop growth. Tilled soil on the slope could increase runoff and erosion rates during heavy rains, in particular when the canopy is not incomplete (Fig 6). As a result, increased runoff and erosion at the field scale could lead to a failure of soil conservation measures at the watershed level. In a study in Canada, Chow et al. [3] found that increased agricultural intensity was linearly related to an increase in runoff and erosion rates at the watershed level. Our plot-based long-term observation in this study positively verifies their observational study experimentally.

### Role of Chinese pine on soil and water conservation

The erosion rate of the Chinese pine plots was significantly lower than that of the wheat plots, which is in good agreement with previous studies [24,26,50]. As one of the main tree species for GGP, Chinese pine reduced runoff and erosion with the increase of the area of GGP, and the erosion reduction is about 30% more than the runoff reduction [50]. From this study, based on our long-term observation, erosion reduction is 48% more than runoff reduction. In our study, the average erosion rate of Chinese pine plots was 0.2 t ha^-1^, which was a reduction of 0.4 t ha^-1^ compared to the 0.6 t ha^-1^ of the wheat plots regardless of slope. Since the year 2000, ecological restoration of the study area was implemented, a large area of farmland characterized by high slopes with vulnerable soils and severe water and soil erosion were switched to afforestation and grassland land uses by planting Chinese pine tree and growing artificial grassland, which resulted in a protected land area of 8.6×10^6^ ha [23]. If using the reduced erosion rate by forest from our study, it is estimated that the soil loss may be reduced by 3.1×10^7^ t due to the afforestation of Chinese pine plantations. In addition to the direct reduction of soil loss, the reduction in sediment yield could also result in a reduction of the organic loss because the loss of organic carbon is overwhelmingly caused by runoff sediment transport (99%) [23]. This will benefit the environmental management of the overall ecosystem in this region.

### Effects of potential explanatory variables on runoff and erosion rates in Chinese pine land

For Chinese pine plots, the runoff rate, erosion rate and erosive rainfall were all positively correlated in our study, which does not agree with the negative correlation between erosive rainfall and erosion rate in a previous study by Maetens et al. [7] in Europe and the Mediterranean. This discrepancy may mainly be attributed to the different geographical context underpinning erosion and runoff processes. Our study area was located in the hilly gully area of the Loess Plateau in China with no land management practices on plots while the study by Maetens et al. [7] was derived from many plot experiments with different land management measures.

The explanatory factors in this study accounted for a relatively large part of the variation in runoff rate and erosion rate, 86–91% of the total variance in runoff erosion rate in pine plots and 74–83% in wheat plots (Table 7) although there was a relatively poorer prediction of runoff rate. When slope was considered, the explained variances were from 66–71% and 86–93% for runoff rate and erosion rate, respectively (Table 8). This may suggest that other influential factors (e.g., season change) beyond those included in this study affected runoff and erosion reduction, especially runoff rate reduction. In turn, if the results from this study are scaled up to a landscape level which may include more impacting factors such as slope length, interactions among various factors, and soil and water conservation measures, the explained variance could be changed by those factors. For example, some previous studies conducted in the Zulihe River, the lower tributary of the studied watershed, reported that 52% to 54% of the variations in runoff and erosion could be captured by rainfall, different soil, and water-and-soil protection measures (number of terraces, woodlands, grasslands, natural preserve, and check dams) [36]. The study by Zhao et al. [40] also showed that the combined effects of rainfall, slope length, slope, and runoff in different farming methods (contour-line farming, grassland cover, hedges, no-till, horizontal terraces) could explain 5–65%, 14–73% of runoff and erosion rates, respectively. According to our study, with certain control measures adopted in the study area, the runoff and soil loss may be reduced but they can only be reduced to the certain level which was still an order of magnitude higher than the initial value. The similar results were reported in other study [9]. Under extreme rainfall conditions, the study area not only experienced an extreme rainstorm event, but sometimes also a rainfall event with low intensity and long duration [51]. The low intensity and long duration rainfall could lead to similar effects of flood on soil erosion process [51]. The relationship between runoff and erosion observed in average or low rainfall may change due to flooding effects, which will increase sediment yield and lead to erosion intensification. Therefore, watershed managers must prepare to minimize the potential severe effects of extreme rainfall [52], with a consideration of slope at landscape level regardless of land use, particularly on steep slopes [51]. However, it should be noted that our study findings from plot scale may be biased when used at the landscape level, as the previous study suggested by [40]. In other words, if the current study or modeling results are intended to be used in modeling large-scale soil loss, a proper scale-up approach must be developed and tested.

### Future study suggestion

While our results should be generally applicable for similar environmental conditions, it should be noted that our experiment only focused on Chinese pine and wheat crop. When other tree species and crops are involved, the reduction rates in soil loss and carbon organic matter may not be at the same rate because different crops and tree species have different canopies and root system structures which are different in their soil and nutrient holding capacity [23,53]. However, the observed reduction pattern of soil and water loss from this study may be applicable under different climatic conditions with some verification. In addition, if engineering measures (e.g. slope-separate flat terraces and cone-shape focused fluid pits) are adopted in the early stage of afforestation [35], soil erosion can be further reduced, and water retention and accumulation in first place can be greatly enhanced, thereby changing the runoff and erosion patterns, which may lead to some discrepancy from the current study. Therefore, a future study focusing on watershed scale modelling is recommended.

## Conclusions

Based on our long-term observation of runoff and erosion plot data, this study quantitatively evaluated the effect of Chinese pine (*Pinus tabulaeformis*) plantations in reducing runoff and erosion in the Semi-arid region in comparison with the wheat plots. The results showed that runoff and erosion rate both increased with the increase of slope regardless of forest or agricultural land use. However, compared with wheat cropping, the runoff rate was reduced by 6–17% and the erosion rate by 48–72% under Chinese pine forest land for a slope range of 10° to 20°. This experimental study illustrated the significance of many long-term ecological and environmental restoring and re-establishing programs such as the policy of returning farmland to forest initialized by the governments in China.

For a given slope condition, erosion rates were better explained than runoff rates by the potential explanatory variables (erosive rainfall, runoff rate). Multiple regression models can be well fitted for the erosion rates of the Chinese pine and wheat plots at all slopes. The explained variance of the erosion rate of Chinese pine plots is 86–91%, higher than 74–83% of wheat plots. After introducing slope as a factor, both runoff rate and erosion rate can be well modeled with linear models regardless of land use. With the current model structures, they can be easily adapted to estimating runoff and erosion rates at the landscape level by scaling up with slope and land use.
